# Supplementary material for: Causal interplay between lactose intolerance and gut microbiota: a combined bidirectional Mendelian randomization and in vivo validation study
Source: Front Nutr. 2026 Jun 1;13:1803337. doi: 10.3389/fnut.2026.1803337 (PMC13265576; doi:10.3389/fnut.2026.1803337)
Supplement: Supplementary file 1 [file Data_Sheet_1.zip › supplementary materials/Forward/leave one out cross validation/ebi-a-GCST90027824.finngen_R12_E4_LACTONAS.pdf]

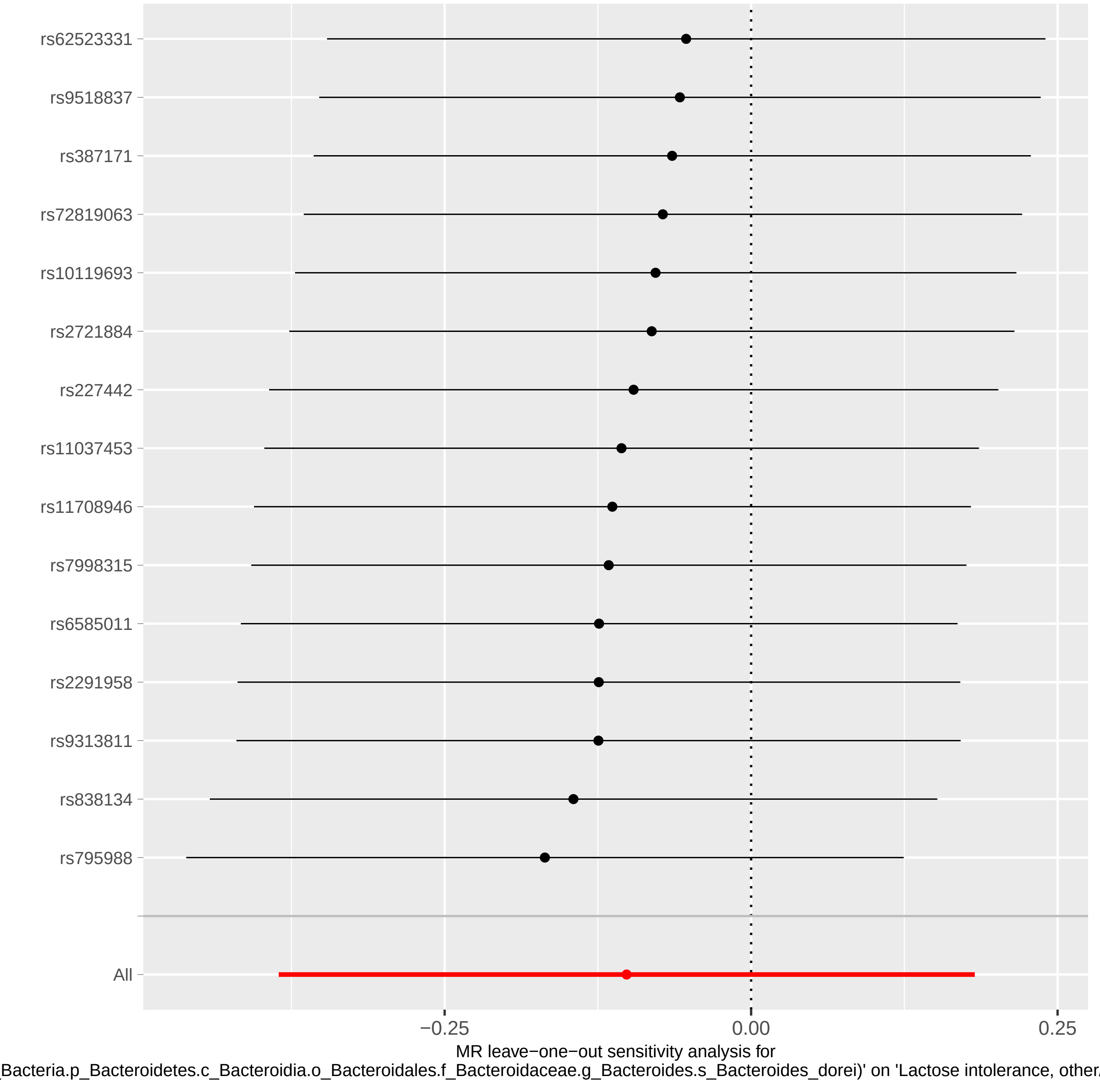

rs62523331
rs9518837
rs387171
rs72819063
rs10119693
rs2721884
rs227442
rs11037453
rs11708946
rs7998315
rs6585011
rs2291958
rs9313811
rs838134
rs795988
All
−0.25
0.00
0.25
MR leave−one−out sensitivity analysis for
_Bacteria.p_Bacteroidetes.c_Bacteroidia.o_Bacteroidales.f_Bacteroidaceae.g_Bacteroides.s_Bacteroides_dorei)' on 'Lactose intolerance, other
